# Gene Transfer Using Micellar Nanovectors Inhibits Choroidal Neovascularization In Vivo

**DOI:** 10.1371/journal.pone.0028560

**Published:** 2011-12-05

**Authors:** Aya Iriyama, Makoto Oba, Takehiko Ishii, Nobuhiro Nishiyama, Kazunori Kataoka, Yasuhiro Tamaki, Yasuo Yanagi

**Affiliations:** 1 Tokyo Metropolitan Geriatric Hospital, Itabashi, Tokyo, Japan; 2 Department of Ophthalmology, University of Tokyo School of Medicine, Bunkyo, Tokyo, Japan; 3 Department of Clinical Vascular Regeneration, Graduate School of Medicine, The University of Tokyo, Bunkyo, Tokyo, Japan; 4 Department of Bioengineering, Graduate School of Engineering, The University of Tokyo, Bunkyo, Tokyo, Japan; 5 Center for Disease Biology and Integrative Medicine, Graduate School of Medicine, The University of Tokyo, Bunkyo, Tokyo, Japan; 6 Department of Materials Engineering, Graduate School of Engineering, The University of Tokyo, Bunkyo, Tokyo, Japan; The University of Hong Kong, Hong Kong

## Abstract

**Purpose:**

Age-related macular degeneration caused by choroidal neovascularization (CNV) remains difficult to be treated despite the recent advent of several treatment options. In this study, we investigated the in vivo angiogenic control by intravenous injection of polyion complex (PIC) micelle encapsulating plasmid DNA (pDNA) using a mice CNV model.

**Methods:**

The transfection efficiency of the PIC micelle was investigated using the laser-induced CNV in eight-week-old male C57 BJ/6 mice. Firstly, each mouse received intravenous injection of micelle encapsulating pDNA of Yellow Fluorescent Protein (pYFP) on days 1,3 and 5. The expression of YFP was analyzed using fluorescein microscopy and western blotting analysis. In the next experiments, each mouse received intravenous injection of micelle encapsulating pDNA of soluble Fms-like tyrosine kinase-1 (psFlt-1) 1,3 and 5 days after the induction of CNV and the CNV lesion was analyzed by choroidal flatmounts on day 7.

**Results:**

Fluorescein microscopy and western blotting analysis revealed that the expression of YFP was confirmed in the CNV area after injection of the PIC micelle, but the expression was not detected neither in mice that received naked pDNA nor those without CNV. Furthermore, the CNV area in the mice that received intravenous injection of the psFlt-1-encapsulated PIC micelle was significantly reduced by 65% compared to that in control mice (p<0.01).

**Conclusions:**

Transfection of sFlt-1 with the PIC micelle by intravenous injection to mice CNV models showed significant inhibition of CNV. The current results revealed the significant potential of nonviral gene therapy for regulation of CNV using the PIC micelle encapsulating pDNA.

## Introduction

Age-related macular degeneration (AMD) is a leading cause of legal blindness in developed countries, and even with the recent advent of several treatment options, treatment of AMD remains difficult [1]–[2]. Vision loss in AMD occurs with the advance of AMD, that is, “exudative” AMD and “geographic atrophy”. Visual loss in exudative AMD is caused by choroidal neovascularization (CNV), i.e., the neovascular vessels extending from the choroid underneath the sensory retina, and the subsequent atrophy of the retinal pigment epithelium (RPE). One of the major factors that induce CNV is vascular endothelial growth factor-A (VEGF-A), a diffusible cytokine that promotes angiogenesis and vascular permeability [3]. Clinical studies have revealed that the intravitreal administration of VEGF-A antagonists such as ranibizumab and bevacizumab, and an RNA aptamer that specifically inhibits the VEGF 165 isoform, i.e., pegaptanib, arrests CNV progression and leakage, and ameliorates exudative change and improves visual acuity [4], [5], [6] However, these drugs need to be used repeatedly at 4- to 6-week intervals [4], [5], [6], which raises concerns about injection-related adverse events, including ocular inflammation, retinal injury, and endophthalmitis.

Another major approach to inhibit the VEGF signaling pathway in CNV is the use of VEGF kinase inhibitors; however, most of the currently developed receptor tyrosine kinase (RTK) inhibitors are not VEGF-selective and also inhibits other RTKs, raising the possibility of unexpected side effects [7]. A previous study from our laboratory has demonstrated that highly VEGF-selective RTK inhibitors are effective in reducing the size of CNV model; however, the study demonstrated that systemic administration of VEGF-selective inhibitors may also lead to unexpected systemic side effects [8]. Another approach to inhibit VEGF signal is the application of soluble VEGF receptor 1 (soluble fms-like tyrosine kinase-1, sFlt-1). sFlt-1 is a potent endogenous molecule and is highly specific to VEGF, and binds VEGF with the same affinity and inhibits its signal transduction [9], [10], [11].

Previous studies from our laboratory and some other groups have demonstrated that macromolecules accumulate to CNV lesion with high efficiency through enhanced permeability and retention (EPR) effect after intravenous injection [12]–[13]. As a first step to develop a drug delivery system utilizing the EPR effect, our group have demonstrated that biocompatible core-shell type nanocarriers, i.e., polyion complex (PIC) micelle formed through the electrostatic interaction between oppositely charged macromolecules, can achieve effective accumulation in the CNV lesion in a mouse model [12], [14]. Moreover, as a promising non-viral vector for gene therapy, PIC micelles consisting of plasmid DNA and poly(ethylene glycol)-*b*-poly{*N*-[*N*-(2-aminoethyl)-2-aminoethyl]aspartamide} block copolymers [PEG-*b*-PAsp(DET)], which show minimal cytotoxicity and high transfection efficiency both in vitro and in vivo [15], [16], [17], [18], [19], have been shown to be utilized for the gene therapy against a mouse corneal neovascularization model by local administration of plasmid encoding sFlt-1 [20].

In this study, we have applied the PIC micelles formed from pDNA and the mixture of PEG-*b*-PAsp(DET) block copolymers and PAsp(DET) homopolymers to the systemic injection to a mice CNV model. Because repeated intraocular injections poses the risks of retinal detachment and endophthalmitis on the patients, a novel strategy to avoid repeated injection is needed. The current study demonstrates an intriguing possibility that systemic administration of the PIC micelles can achieve effective and safe treatment of CNV.

## Results

### Targeted gene expression in the CNV area after intravenous injection of pYFP-PM

**Figure 1:**
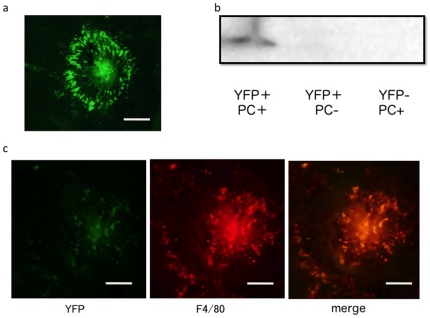
Preparation of PIC micelles encapsulating pDNA.

Firstly, experiments were performed to determine the efficacy of gene expression of intravenous injection of pYFP-PM. The CNV lesions were induced by laser photocoagulation. Subsequently, each mouse received an intravenous injection of 200 µL of pYFP-PM or PBS, and the expression pattern was evaluated by fluorescent microscopy. At seven days after the injection, choroidal flatmount and histological analysis demonstrated YFP fluorescence in the CNV area after intravenous injection of pYFP-PM (Fig1 a). Furthermore, Western blotting analysis demonstrated that YFP protein was detected in the eyes with CNV, after intravenous injection of pYFP-PM. YFP expression was detected neither after generation of CNV alone nor after intravenous injection of pYFP-PM without CNV (Fig1 b). Next, immunohistochemistry was performed to examine the cells in which YFP protein expressed. The results demonstrated that the localization of F4/80 was partially overlapping the expression of YFP, indicating that YFP protein expressed in F4/80-positive macrophage (Fig1 c).

### Intravenous injection of psFlt-1-PM decreased the area of CNV

**Figure 2:**
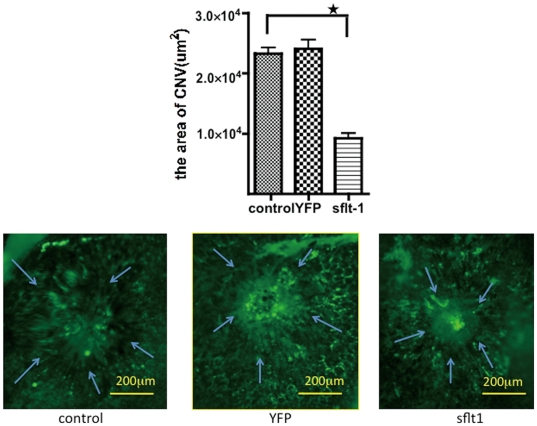
Targeted gene expression in the CNV area after intravenous injection of pYFP-PM. a: Choroidal flatmount and histological analysis demonstrated YFP fluorescence in the CNV area after intravenous injection of pYFP-PM. b: Western blotting analysis demonstrated that YFP protein was detected in the eyes with CNV, after intravenous injection of pYFP-PM. YFP expression was detected neither after generation of CNV alone nor after intravenous injection of pYFP-PM without CNV. c: The results of immunohistochemistry demonstrated that the expression of F4/80 was partially overlapping the expression of YFP, indicating that YFP protein expressed in F4/80-positive macrophage. CNV: choroidal neovascularization, pYFP-PM: the PIC micelles encapsulating pYFP (yellow fluorescent protein), PC: photocoagulation, bar:100 um.

The results led us to investigate the effects of the transfection of the plasmid encoding human sFlt-1 in CNV. After CNV was induced, mice received an intravenous injection of psFlt-1-PM or pYFP-PM and PBS. Quantification of the CNV lesion demonstrated that whereas the administration of pYFP-PM had no significant effects on the area, the neovascularized area in the mice that received an intravenous injection of psFlt-1-PM was significantly reduced by 60% than that of control mice (n = 7, p<0.01) (Fig 2). In addition, no remarkable abnormality in the fundus, other than at the photocoagulated sites was detected.

### Systemic adverse effect

We investigated the systemic effects caused by intravenous injection of the PIC micelles. Laboratory data, including aspartate aminotransferase (AST), alanine aminotransferase (ALT), blood urea nitrogen (BUN), and creatinine were within normal limits after the venous injection of the PIC micelles (Table1).

**Table 1:** Laboratory data.

	AST(IU/L)	ALT(IU/L)	BUN(mg/dL)	creatinine(mg/dL)
control	58.7±5.7	24.0±3.1	29.6±2.6	0.21±0.01
PIC micelle	63.4±4.8	25.0±1.4	25.6±1.5	0.19±0.02

## Discussion

PEG-*b*-PAsp(DET) block copolymers show a remarkably high transfection efficacy and low toxicity [15]. The PIC micelles are substantially stable in vivo, because of their characteristic core-shell structure consisting of the plasmid DNA-loaded PIC core surrounded by the PEG shell with the sterically repulsive propensity. Moreover, after internalization into cellular compartments, PEG-*b*-PAsp(DET) is expected to facilitate efficient translocation of the micelle toward the cytoplasm since the ethylenediamine side chain of PAsp(DET) might exhibit a low pH-selective membrane disruptive function^ 4^. In the current study, to investigate its potential for anti-angiogenic therapy, we have developed the PIC micelles composed of pDNA and the mixture of PEG-*b*-PAsp(DET) block copolymers and PAsp(DET) homopolymers for systemic gene delivery, and demonstrated that transgene expression is achieved by intravenous injection of the PIC micelles in CNV area. Furthermore, we demonstrated that transfection of the PIC micelles encapsulating psFlt-1 by intravenous injection to mice CNV models showed significant inhibition of developing CNV.

Recently, it has been demonstrated that intravitreous injection of VEGF-A antagonists such as ranibizumab, bevacizumab, and pegaptanib arrests CNV progression and leakage from CNV, and improves visual acuity [21]–[24]. However, these drugs need to be used repeatedly at 4- to 6-week intervals, which raises concerns about injection-related adverse events including ocular inflammation, retinal injury, and endophthalmitis. Intravitreal adeno-associated virus (AAV) expressing sFlt-1 can achieve prolonged expression in mice; however, intraocular injection of AAV was associated with vitreal inflammation [25]. Furthermore the safety of long-term intraocular VEGF-A neutralization remains unknown [26], [27]. Previously we have evaluated the effects of bevacizumab and anti-rat VEGF antibody on retinal ganglion cells both in vivo and in vitro [28]. The results demonstrated that the short term VEGF-A neutralization showed no toxicity on retinal ganglion cells. However, another group reported that 6-month sustained expression of sFlt-1 in retinal pigment epithelial cells in mice results in photoreceptor degeneration [29]. Furthermore, several investigations report the reduction in retinal vessel diameter and flow velocities in eyes with exudative AMD 3 months after intravitreal anti-VEGF antibody treatment [30], [31], [32]. In this study, we demonstrated that the gene expression was seen only in CNV area after intravenous injection of the PIC micelles. Furthermore there were no apparent histological abnormalities after intravenous injection of the PIC micelles. Moreover, laboratory data showed no abnormal values in physiological parameters under the current condition. This is in contrast to our previous study that used systemic administration of VEGF-selective RTK inhibitors in CNV model [33]. Thus, the current results support that the nonviral gene therapy for regulation of CNV using the PIC micelles have a great advantage in several points. Firstly, intravenous injection deemed to be free of ocular complications. Secondly, the gene expression is limited to CNV area and the effect of long term VEGF-A neutralization on retina is reduced compared to the current therapy, i.e., intravitreous injection of VEGF antagonist. Disruption of VEGF signaling might be effective for CNV treatment. However, it may also inhibit angiogenesis that is needed for vascular maintenance and repair under normal conditions angiogenesis. Therefore, even if blockage of VEGF signaling is effective in suppressing CNV development, inhibition of VEGF signaling in the retina and other organs, particularly those of advanced age, such as AMD patients, may in fact be dangerous. In turn, our system presented here clearly demonstrated target-tissue specific expression of pDNA, and is expected to be used without systemic adverse effects as described above. It is generally accepted that blockade of VEGF signaling pathway leads to decreased macrophage infiltration, although further studies are needed.

To put this treatment to clinically use, it is important to investigate the most appropriate dose and dosing regimen, although the current study employed multiple injection based on our preliminary results and considering the fact that expression level of the transferred gene was low when compared to viral vectors.

In conclusion, we have explored the possibility of the treatment of exudative AMD via systemic gene delivery utilizing the PIC micelles encapsulating plasmid DNA. The obtained results have demonstrated that the PIC micelles accumulated in CNV, thereby exhibiting high therapeutic efficiency against mouse AMD model. This study suggests the current system that blocks local VEGF by the systemic gene therapy using the PIC micelles encapsulating sFlt-1 has a potential as a novel anti-angiogenic treatment for CNV.

## Materials and Methods

### Animals

Eight-week-old male C57 BJ/6 mice were purchased from Saitama Laboratory Animal Supply Inc. (Saitama, Japan). All animal experiments conformed to the ARVO Statement for the Use of Animals in Ophthalmic and Vision Research for animal use. All animal experiments were carried out in accordance with the guidelines for animal experiments at the University of Tokyo, Japan, and were approved by the Animal Care Committee of the University of Tokyo. (approval No. med-p10-020)

All procedures were performed with the animals under general anesthesia by administration of mixture (1.5 ml/kg) of keramin hydrochloride (Ketalar, Sankyo, Tokyo, Japan) and xylazine hydrochloride (Celactal, Bayer, Tokyo, Japan).

### Preparation of PIC micelles encapsulating pDNA

**Figure 3:**
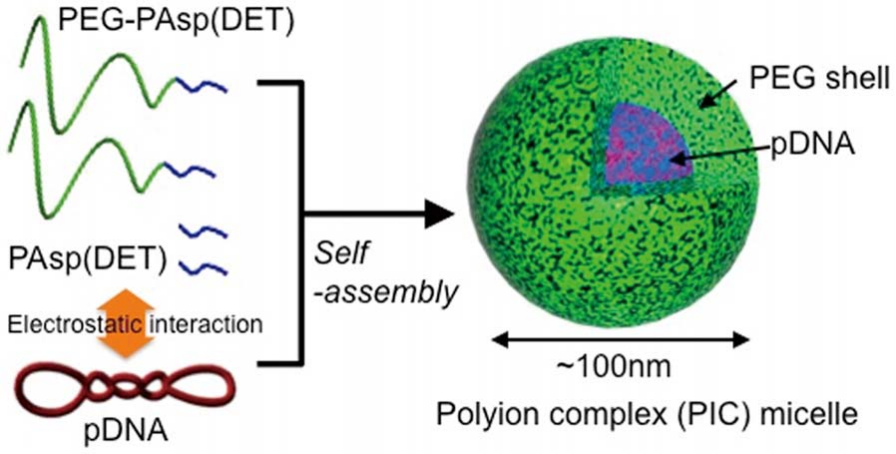
Intravenous injection of psFlt-1-PM decreased the area of CNV. Quantification of the CNV lesion demonstrated that the neovascularized area in the mice that received psFlt-1-PM was significantly reduced by 60% than that of control mice (n = 7, p<0.01), whereas the administration of pYFP-PM had no significant effects on the neovascularized area. The lower panels show representative micrographs. Arrows indicate the CNV lesion. CNV: choroidal neovascularization, psFlt-1-PM: the PIC micelle encapsulating psFlt-1 (fms-like tyrosine kinase-1), pYFP-PM: the PIC micelles encapsulating pYFP (yellow fluorescent protein).

A fragment cDNA of SEYFP-F46L, a variant of a yellow fluorescent protein with the mutation F46L, provided by RIKEN (Wako, Japan), was inserted into the pCAcc vector (pYFP) and a fragment cDNA of human soluble Flt-1, provided by Dr. Masashi Shibuya, was inserted into the pVL1393 vector (psFlt-1). PEG-*b*-PAsp(DET) block copolymers and PAsp(DET) homopolymers were synthesized as previously reported [15], [16], [17]. PIC micelles encapsulating pDNA were prepared by mixing pDNA and a mixture of PEG-*b*-PAsp(DET) and PAsp(DET) in 10 mM Hepes buffer (pH 7.3) at the block copolymer/homopolymer ratio of 70/30 on the basis of amine numbers and the N/P ratio (N =  total amines in polycations; P =  total phosphate anions in pDNA) of 8 (pDNA concentration: 100 µg/mL) (Fig3).

### Generation of CNV by laser photocoagulation

After the pupil was dilated with 1 drop of 0.5% tropicamide (Mydrin M; Santen Pharmaceutical, Osaka, Japan), the CNV lesions were induced by laser photocoagulation as has been previously described [34]. A glass cover served as a contact lens. Diode-laser (DC-3000, NIDEK, Osaka, Japan) and irradiation was delivered through a slit lamp (SL150, Topcon, Tokyo, Japan). The following laser parameters were used; spot size of 200 mm, power of 100 mW, and exposure duration of 20 msec. One and four lesions were created between the major retinal vessels in each fundus, to test transfection efficiency and treatment efficacy, respectively. To test transfection efficacy, mice were assigned to control or treatment group and received intravenous injection of the PIC micelles encapsulating pYFP (pYFP-PM; n = 10 in each group). Each mouse received an intravenous injection of 200 µL of pYFP-PM or PBS 1, 3 and 5 days after laser photocoagulation. Seven days after laser photocoagulation, the tansfection efficacy was investigated by fluorescent microscopy and Western blot analyses. For the analysis of the efficacy of the PIC micelles encapsulating psFlt-1 (psFlt-1-PM), twenty mice (n = 7 in each group) were randomly assigned to psFlt-1-PM, or pYFP-PM or control group. Each mouse received a intravenous injection of 200 µL of psFlt-1-PM, pYFP-PM or PBS 1, 3 and 5 days after laser photocoagulation. CNV lesion size was investigated by choroidal flatmount analysis.

### In vivo detection of YFP

To investigate the transfection efficacy of the PIC micelles, expression of YFP was examined by fluorescent microscopy using RPE/choroidal flatmount analysis. The eyes were enucleated, and cornea, lens, and vitreous were removed and the RPE/choroid was separated and four radial cuts were made at the peripheral choroid to the equator, and the RPE/choroid was flatmounted on a slide with the vitreous side facing up. The flatmount were viewed by fluorescent microscope with an YFP selective filter set (excitation wavelength 500 nm, filter wavelength 530 nm). Photographs were taken with the aid of a CCD camera. Samples from mice with CNV but without treatment served as negative controls, and showed no fluorescence under the current conditions.

### Immunostaining

Immunostaining was preformed essentially as described previously [34]. Briefly, seven days after laser photocoagulation, the RPE-choroidal flatmount was made, and the sample was incubated with rabbit polyclonal antibody against anti-F4/80 (Abcam) at a concentration of 1∶100. Alexa525 conjugated secondary antibody was used to visualize the immunostaining. The samples were viewed with fluorescence microscope.

### Western-blot analysis

Seven days after laser photocoagulation, the RPE/choroid samples were lysed with ice-cold RIPA buffer (50 mM Tris-HCL (PH 7.4), 1% NP-40, 0.25% Na-deoxycholate, 150 mM NaCl, and 1 mM EDTA). The lysates were centrifuged for 15 min at 14000rpm in a microcentrifuge. Protein content of the resultant supernatants was measured by using BCA Protein assay kit. Protein samples were dissolved in sample buffer at a concentration of 1∶1 and were resolved by SDS-PAGE and electroblotted onto a polyvinylidene difluoride membrane. The membrane was placed in blocking solution (5% nonfat dry milk in TBST) for one hour at room temperature. The membranes were incubated overnight at 4°C with anti-YFP antibody (Abcam) at a concentration of 1∶100. The membranes were incubated with horseradish peroxidase labeled second antibody (Amersham biosciences) at a concentration of 1∶5000 for one hour at room temperature and were developed with ECL Plus Western Blotting Detection Reagents (General Electric Company).

### Quantitative analysis of the CNV lesions

Choroidal flatmount analysis was performed to quantify the CNV lesions and CNV was imaged by lectin angiography as has been described elsewhere [34]. Mice received intravenous BS-1 lectin conjugated with FITC (Vector Laboratories, Burlingame, CA) seven days after laser photocoagulation. Then the eyes were enucleated, and the RPE/choroid samples prepared with aforementioned method were flatmounted on a slide with the vitreous side facing up. NIH Image was used for the image analysis. The quantification of the CNV was performed in a masked manner.

### Statistical Analysis

The one-way analysis of variance (ANOVA) was used to compare the CNV area. P values of less than 0.05 were considered significant.
